# Adenine base editing rescues pathogenic phenotypes in tissue engineered vascular model of Hutchinson-Gilford progeria syndrome

**DOI:** 10.1063/5.0244026

**Published:** 2025-02-26

**Authors:** Nadia O. Abutaleb, Xin D. Gao, Akhil Bedapudi, Leandro Choi, Kevin L. Shores, Crystal Kennedy, Jordyn E. Duby, Kan Cao, David R. Liu, George A. Truskey

**Affiliations:** 1 Department of Biomedical Engineering, Duke University, Durham, North Carolina 27708, USA; 2 Merkin Institute of Transformative Technologies in Healthcare, Broad Institute of MIT and Harvard, Cambridge, Massachusetts 02142, USA; 3 Department of Chemistry and Chemical Biology, Harvard University, Cambridge, Massachusetts 02138, USA; 4 Howard Hughes Medical Institute, Harvard University, Cambridge, Massachusetts 02138, USA; 5 University Program in Genetics and Genomics, Duke University, Durham, North Carolina 27708, USA; 6 Department of Cell Biology and Molecular Genetics, University of Maryland, College Park, Maryland 20742, USA

## Abstract

The rare, accelerated aging disease Hutchinson-Gilford Progeria Syndrome (HGPS) is commonly caused by a *de novo* c.1824 C > T point mutation of the *LMNA* gene that results in the protein progerin. The primary cause of death is a heart attack or stroke arising from atherosclerosis. A characteristic feature of HGPS arteries is loss of smooth muscle cells. An adenine base editor (ABE7.10max) corrected the point mutation and produced significant improvement in HGPS mouse lifespan, vascular smooth muscle cell density, and adventitial fibrosis. To assess whether base editing correction of human HGPS tissue engineered blood vessels (TEBVs) prevents the HGPS vascular phenotype and to identify the minimum fraction of edited smooth muscle cells needed to effect such changes, we transduced HGPS iPSCs with lentivirus containing ABE7.10max. Endothelial cells (viECs) and smooth muscle cells (viSMCs) obtained by differentiation of edited HGPS iPSCs did not express progerin and had double-stranded DNA breaks and reactive oxygen species at the same levels as healthy viSMCs and viECs. Editing HGPSviECs restored a normal response to shear stress. Normal vasodilation and viSMC density were restored in TEBVs made with edited cells. When TEBVs were prepared with at least 50% edited smooth muscle cells, viSMC proliferation and myosin heavy chain levels significantly improved. Sequencing of TEBV cells after perfusion indicated an enrichment of edited cells after 5 weeks of perfusion when they comprised 50% of the initial number of cells in the TEBVs. Thus, base editing correction of a fraction of HGPS vascular cells improves human TEBV phenotype.

## INTRODUCTION

The rare and fatal genetic disease Hutchinson-Gilford Progeria Syndrome (HGPS) causes accelerated aging.^1^ A *de novo* point mutation of the LMNA gene (c.1824 C > T) results in a truncated and farnesylated form of lamin A, known as progerin.^1^ Progerin accumulates in the nuclear lamina, causing progressive damage with aging,^2^ including increased DNA double-stranded breaks (DSB), aberrant nuclear shape and blebbing, changes in gene expression, heterochromatin loss, telomere shortening, and cellular senescence.^1,2^ Pathologies exhibited by individuals with HGPS include lack of subcutaneous fat, reduced size, bone abnormalities, hair loss, and cardiovascular disease.^1,3,4^ Severe progressive atherosclerosis in HGPS patients causes death by heart attack or stroke.^5^

Strategies to treat HGPS include correcting the mutation, blocking progerin synthesis or function, increasing progerin removal, or altering lamin A splicing.^2^ Lonafarnib, a farnesyltransferase inhibitor, is currently the only treatment approved by the FDA. Lonafarnib decreases progerin in the nuclear membrane^2,6^ and improves several HGPS symptoms including cell nuclear morphology.^3,7–11^ In animal models, lonafarnib extended lifespan, improved bone development, increased body weight and the amount of adipose tissue, and reduced vascular stiffness.^2,12–15^ In HGPS patients, lonafarnib treatment improved weight and bone density and reduced cardiovascular stiffness, resulting in a modest decrease in mortality.^3,6,16^ Given the modest increase in lifespan, efforts continue to develop a therapy to eliminate or drastically reduce HGPS symptoms.

The ideal therapy for a disease caused by a spontaneous heterozygous point mutation like HGPS would correct the mutated allele back to wild type. However, using CRISPR-Cas9 to generate a point mutation can produce undesirable insertions and deletions (indels), translocations, and rearrangements as cells respond to the DSB created by Cas9.^17^ Two mouse studies showed reduced progerin expression by eliminating *LMNA* expression entirely with CRISPR-Cas9. This change improved body weight, epidermal thickness, and lifespan despite low editing efficiency.^18,19^ However, the cardiovascular benefit was low, with only one study showing an improvement in aortic SMC density.^18^ Additionally, while the loss of lamin A in mice has no adverse effect, the impact of lamin A deletion in humans is not known.^19^

Base editing is a novel approach to generate precise point mutations using Cas9 nickase tethered with natural or lab-directed deaminase domain by directly converting a single DNA base without generating DSBs.^17^ This technique has been used to correct mutations relating to muscular dystrophy, HGPS, sickle cell disease, and spinal muscular atrophy in mouse models.^20–23^ Adenine base editing (ABE) to change the mutated A•T base pair in *LMNA* Exon 11 to wild-type G•C could potentially correct HGPS with a single-dose therapy. ABE treatment on HGPS fibroblasts and progeroid mice produced much lower progerin protein levels and fewer abnormally shaped nuclei in HGPS fibroblasts and improved vascular SMC density and adventitial fibrosis in progeroid mice.^21^ Another study testing ABE treatment on HGPS iPSC-derived endothelial cells showed improved HGPS viEC tube formation and nitric oxide production under static conditions with treatment.^24^ The effects of ABE treatment on HGPS ECs under physiological flow conditions and the ABE effect on other vascular phenotypes as well as human blood vessels, including function and fibrosis, have not been reported.

To model progeria *in vitro*, we fabricated human tissue engineered blood vessels (TEBVs) from endothelial cells (viECs) and smooth muscle cells (viSMCs) derived from induced pluripotent stem cells (iPSCs) of HGPS and healthy individuals.^25^ This model reproduced key features of the vascular pathology, including thicker TEBVs, reduced vasoactivity, number of smooth muscle cells per unit cross-sectional area, calcification, increased extracellular matrix, and inflammation. The treatment of HGPS TEBVs with lonafarnib for 1 week reduced these pathological features. Treating HGPS TEBVs with lonafarnib and the rapamycin analog Everolimus together led to further improvements in vasoactivity and viSMC and viEC protein expression.^26^ Thus, the HGPS TEBVs represent a suitable human model to test vascular function *in vitro*.

In this study, we used the HGPS TEBV model to test the effects of ABE on viECs and viSMCs derived from iPSCs of individuals with HGPS and TEBVs made with edited and unedited HGPS viECs and viSMCs (supplementary material, Fig. S1). The TEBV platform allows us to identify the fraction of edited cells needed to restore function by evaluating vascular function, cell composition, and structure. Base editing of HGPS iPSCs was used to produce viECs that restored flow responses similar to healthy viECs. TEBVs made with edited cells restored normal vasoactivity, viSMC numbers, and viSMC contractile protein expression. When TEBVs were made with a mixture of edited and unedited cells, viSMC proliferation and myosin heavy chain 11 (MHC11) levels significantly improved relative to HGPS TEBVs.

## RESULTS

### Adenine base editing corrects HGPS mutation in two HGPS cell lines

**FIG. 1.**
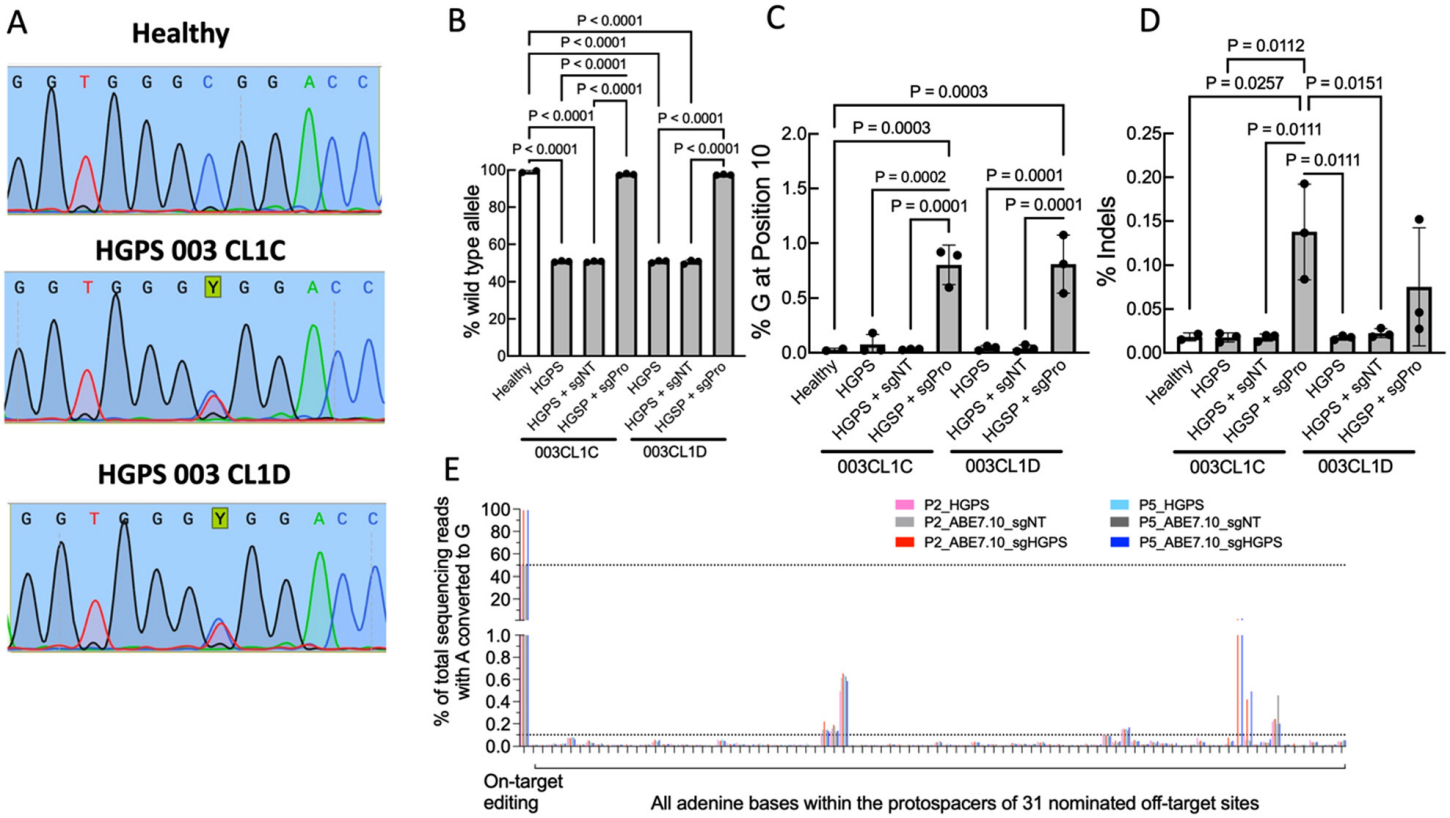
ABE7.10max treatment restores 97% wild-type allele at HGPS mutation site in two HGPS cell lines. (a) Sanger sequencing of Healthy 168 CL2, HGPS 003 CL1C, and HGPS 003 CL1D iPSCs showing heterozygous 1824 C > T mutation in the *LMNA* gene of HGPS cells. (b) Percent wild-type allele at the *LMNA* 1824 C > T mutation site from high throughput sequencing of healthy 168 CL2, HGPS 003 CL1C, and HGPS 003 CL1D iPSCs. HGPS cells were either untreated, treated with ABE7.10max with non-targeting single guide RNA (sgNT), or treated with ABE7.10max with *LMNA* mutation-targeting single guide RNA (sgPro). (c) Percent bystander editing at the A_10_ off-target site and (d) percent indels found in healthy and HGPS untreated and ABE7.10max-treated iPSCs based on high throughput sequencing. Data presented as mean± SD. N = 3 for each group, except for healthy iPSCs for which N = 2. Results were compared using one-way ANOVA followed by a post hoc Tukey's test. (e) DNA sequencing of the top 31 off-target loci identified by Koblan *et al.*^21^ Samples were from passage 2 (P2) and passage 5 (P5) viSMCs.

After optimizing the puromycin (1 *μ*g/ml) and lentivirus doses (0.002 v/v), we tested ABE7.10max (ABE) treatment on two independent clonally expanded iPSC cell lines from the HGPS donor 003 (003 CL1C and 003 CL1D) (Progeria Research Foundation). Sanger Sequencing confirmed the heterozygous *LMNA* 1824 C > T mutation in both HGPS cell lines [Fig. 1(a)]. We assessed treatment editing efficiency, bystander editing, insertion/deletion (indel), and off-target site frequency by high throughput DNA sequencing of untreated and treated iPSCs.

The complementary strand sequence surrounding the mutation is as follows: GGTCC**A_6_**CCC**A_10_**CCTGGGCTCCTGA, where **A_6_** is the editing site for the pathogenic mutation at protospacer position 6, and **A_10_** is a nearby adenine that is not mutated. Editing efficiency was defined as the percent wild-type allele (G) at the **A**_6_ mutation position, and bystander editing was defined as the percent of G at the **A_10_** position. Controls included healthy 168 CL2 iPSCs and a modified ABE treatment with a non-targeting guide RNA (sgNT).^27^ HGPS cells were either left untreated or transduced with lentivirus containing ABE sgNT or ABE with the *LMNA* mutation-targeting guide RNA (sgPro).

Healthy iPSCs exhibited 99.1% wild-type allele at the site of the mutation, while HGPS iPSCs from both cell lines indicate a heterozygous mutation with only 50.9% wild-type allele at the mutation site [Fig. 1(b)]. HGPS iPSCs treated with the sgNT control did not display any increase in percent wild-type allele at the mutation site [Fig. 1(b)]. HGPS iPSCs treated with the sgPro ABE exhibited 97.7% and 97.5% wild-type allele in the 003 CL1C and the 003 Cl1D cell lines, respectively, and did not significantly differ in percent wild-type allele from the healthy donor [Fig. 1(b)]. sgPro treatment resulted in <1% bystander editing [Fig. 1(c)] and <0.15% indel frequency in both cell lines [Fig. 1(d)].

To analyze any off-target editing in the viSMCs (P2 and P5) differentiated from iPSCs transduced with the sgPro ABE, we performed amplicon sequencing of the top 31 adenine off-target sites previously identified by CIRCLE-seq.^21^ The majority of the adenines in the off-target protospacers showed less than 0.1% editing (background level of Miseq detection limit) [Fig. 1(e)]. The untreated P2 and P5 HGPS have less than 0.1% modification, as expected. For the A2, A6, A10 protospacer sites in one of the nominated off-target sites, the editing is non-differentiated (<0.1%) when compared to sgNT to sgPro, indicating that this is just the sequencing-related during Miseq and not off-target editing. The only true off-target editing is in one genomic site with 2.1–2.6% editing upon sgPro treatment. The adenine at the A10 protospacer site has 0.5% editing. The genomic sequence is mapped to a non-coding sequence (hg19 assembly, chr17: 16875518–16875541), which should have no or minimal effects on the cell fitness.

Progerin was not present in viSMCs and viECs differentiated from iPSCs transduced with the sgPro but was present in viSMCs and viECs differentiated from iPSCs transduced with the sgNT (supplementary material, Fig. S2). To confirm that the editing efficiency is maintained in differentiated cells, we performed high throughput DNA sequencing on viECs and viSMCs differentiated from HGPS 003 CL1D iPSCs transduced with sgNT or sgPro ABE. viSMCs differentiated from transduced iPSCs had 50% wild-type allele in sgNT-transduced cells and 99% wild-type allele in sgPro-transduced cells [supplementary material, Fig. S3(a)]. viSMCs differentiated from sgPro-transduced iPSCs also exhibited 1.6% bystander editing and <0.14% indel frequency [supplementary material, Figs. S3(b)–S3(c)]. viECs differentiated from sgNT-transduced HGPS iPSCs showed no change in wild-type allele at the mutation site (51%), while viECs differentiated from sgPro-transduced HGPS iPSCs exhibited 99% wild-type allele at the mutation site (supplementary material, Fig. S3(d)]. viEC bystander editing in sgPro-treated cells was ∼1.6%, and indels remained below 0.15% (supplementary material, Figs. S3(e)–S3(f)]. Subsequently, “sgNT viECs/viSMCs” refers to viECs or viSMCs differentiated from HGPS 003 CL1D iPSCs transduced with ABE + non-targeting RNA, and “sgPro viECs/viSMCs” refers to viECs or viSMCs differentiated from HGPS 003 CL1D iPSCs transduced with ABE + *LMNA* mutation-targeting RNA. Overall, these editing results are similar to what was obtained previously with HGPS donors 167 and 188 fibroblasts.^21^

### Base editing reduces nuclear blebbing, reactive oxygen species (ROS) levels, and DNA damage and increases proliferation in HGPS viSMCs and viECs

**FIG. 2.**
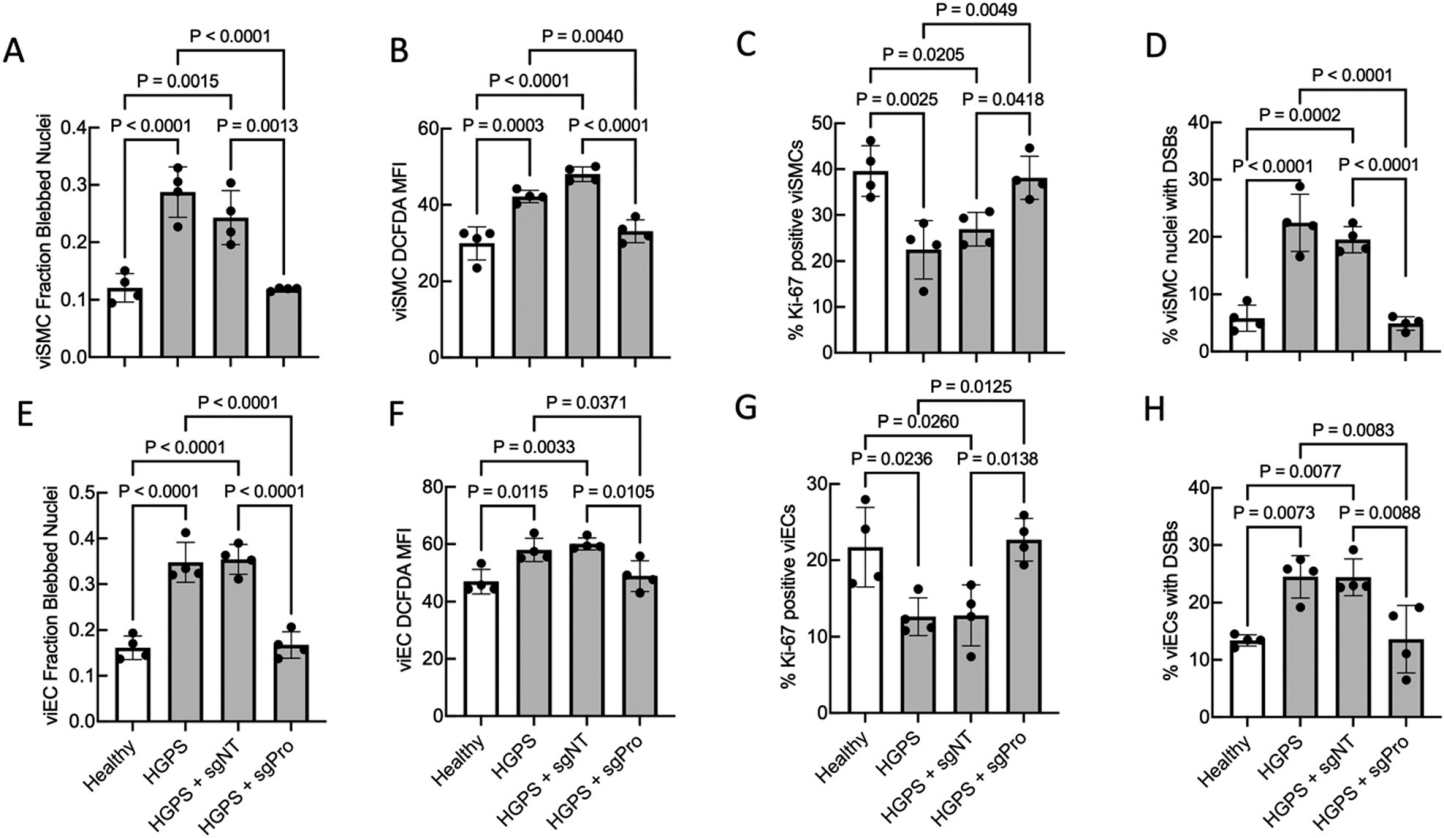
ABE7.10max treatment reduces viSMC (a)–(d) and viEC (e)–(h) nuclear blebbing (a), (e), ROS generation (b), (f), cell proliferation (c), (g), and double-stranded breaks (DSBs) (d), (h). We examined healthy and HGPS viSMCs and viECs derived from iPSCs, which were untreated, treated with ABE7.10max + non-targeting guide RNA (sgNT), or treated with ABE7.10max + *LMNA* mutation-targeting guide RNA (sgPro). Healthy cells are shown as white bars, and HGPS conditions are shown in gray. Results were compared using one-way ANOVA followed by a post hoc Tukey's test. Data are presented as mean ± SD. N = 4 per group.

Similar to our previous results,^25,26^ HGPS viSMCs [Figs. 2(a)–2(d)] and viECs [Figs. 2(e)–2(h)] exhibit increased levels of nuclear blebbing and ROS damage and decreased proliferation compared with healthy cells. The viSMCs and viECs derived from sgNT treated iPSCs displayed similar levels as untreated HGPS cells for nuclear blebbing: DCFDA that measures ROS, Ki67 positive cells that measures proliferation, and percent nuclei positive for γH2A.X that measures double-stranded breaks (DSBs). In contrast, viSMCs and viECs differentiated from iPSCs treated with sgPro displayed significantly lower levels of nuclear blebbing, ROS levels and DSBs, and increased Ki67-positive cells than their untreated HGPS and sgNT counterparts.

Untreated and sgNT-treated HGPS viSMCs and viECs displayed increased DNA damage including an increased percentage of nuclei exhibiting DSBs [Figs. 2(d) and 2(h)] and increased number of nuclear DSBs per total cells [Figs. S4(a) and S4(c)]. sgPro editing reduced the percent of nuclei with DSBs and DSBs per cell to levels present in healthy viSMCs and viECs [Figs. 2(d) and 2(h), supplementary material, Figs. S4(a) and S4(c)]. The number of DNA DSBs per ɣH2A.X-positive nucleus did not significantly change with disease state or treatment condition in viSMCs or viECs [supplementary material, Figs. S4(b) and S4(d)].

### Base editing HGPS viECs restores the physiological response to shear stress

**FIG. 3.**
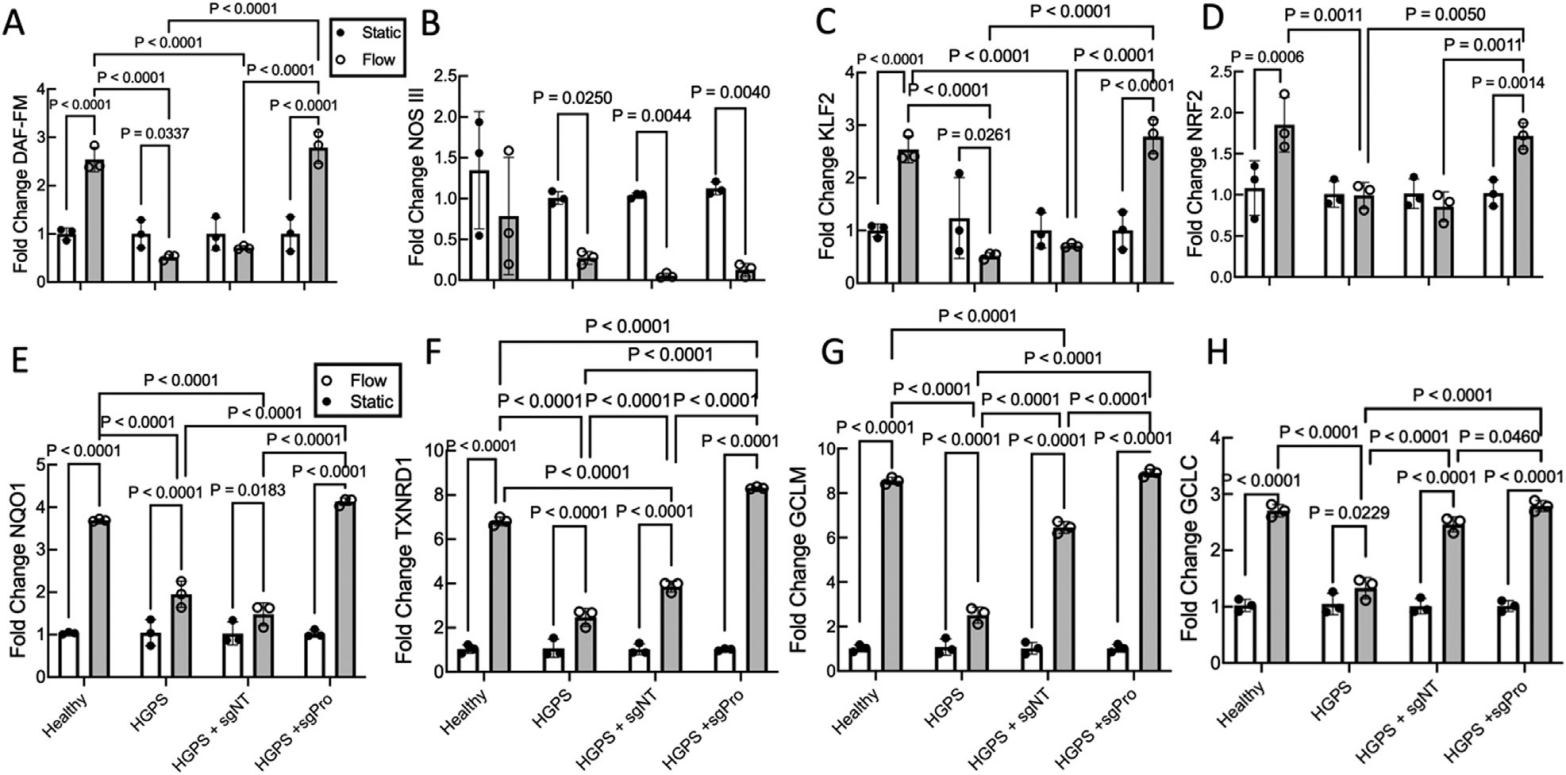
ABE7.10max treatment increases nitric oxide production and flow-mediated gene expression in HGPS viECs exposed to physiological shear stress. (a) DAF-FM diacetate mean fluorescence intensity (MFI) in healthy and HGPS viECs exposed to 12 dynes/cm^2^ shear stress for 24 h. HGPS viECs were either untreated or treated with ABE7.10max + non-targeting guide RNA (sgNT) or ABE7.10max + *LMNA* mutation-targeting guide RNA (sgPro). Data presented as fold change DAF-FM diacetate MFI under shear stress compared with static condition from the same group. (b)–(h) Gene expression of (b) *NOS3*, (c) *KLF2*, (d) *NRF2*, (e) *NQO1*, (f) *TXNRD1*, (g) *GCLM*, and (h) *GCLC* in healthy and HGPS untreated or treated viECs exposed to 12 dynes/cm^2^ shear stress for 24 h. Data were normalized to *GAPDH* expression, and gene expression in viECs exposed to shear stress was referenced to respective viECs under static culture from the same group. Data presented as mean ± SD. N = 3 independent experiments per group. Results compared using two-way ANOVA (flow and cell type treatment) followed by a post hoc Tukey's test.

When exposed to steady shear stress for 24 h, healthy viECs produced higher levels of DAF-FM fluorescence, which indicates nitric oxide (NO) concentration^28^ [Fig. 3(a)]. In contrast, HGPS viECs exposed to shear stress exhibited reduced levels of NO, and sgNT viECs did not exhibit any change in NO production, consistent with dysfunctional endothelium. Edited sgPro viECs had increased levels of DAF-FM after exposure to shear stress that did not differ from healthy viECs. This result indicated that editing HGPS viECs restores shear stress-dependent NO production to normal levels [Fig. 3(a)].

We next examined the effect of editing upon several shear stress-sensitive genes. As we observed previously in healthy viECs,^26^ shear stress did not significantly change *NOS3* mRNA but did increase expression of the flow sensitive transcription factors Kruppel-like factor 2 (*KLF2*), nuclear erythroid 2-related factor 2 (*NRF2*), and *NRF2* sensitive genes NQO1 (NAD(P)H-quinone oxidoreductase 1), *TXNRD1* (thioredoxin reductase 1), *GCLM* (glutamate-cysteine ligase modifier subunit), and *GCLC* (glutamate-cysteine ligase catalytic subunit) [Figs. 3(e)–3(h)]. In response to shear stress, HGPS viECs downregulated *NOS3* and *KLF2* [Figs. 3(b) and 3(c)], and the *NRF2* expression did not significantly change after exposure to flow [Fig. 3(d)]. The shear stress response of NRF2 in HGPS viECs led to significantly smaller increases in NRF2 regulated genes in HGPS viECs [Figs. 3(e)–3(h)].

Transducing HGPS viECs with ABE7.10max and sgNT did not alter the shear stress mediated response of *NOS3*, *KLF2*, *NRF2*, or *NQO1* [Figs. 3(b)–3(e)]. Interestingly, compared with untreated HGPS viECs, sgNT-treated HGPS viECs did express significantly higher levels of *TXNRD1, GCLM*, and *GCLC* after exposure to shear stress, and only *TXNRD1* and *GCLM* gene expression levels were significantly lower than healthy viECs after exposure [Figs. 3(f)–3(h)].

Although sgPro viECs had normal NO production after flow [Fig. 3(a)], sgPro viECs did not affect *NOS3* expression after exposure to shear stress [Fig. 3(b)]. However, sgPro viECs expressed significantly higher levels of *KLF2*, *NRF2*, *TXNRD1*, *NQO1*, *GCLM*, and *GCLC* compared with untreated HGPS viECs after exposure to shear stress [Figs. 3(c)–3(h)]. sgPro viECs restored normal expression of *KLF2*, *NRF2*, *TXNRD1*, *GCLM*, and *GCLC* after exposure to shear stress [Figs. 3(c)–3(h)].

### Base editing restores HGPS TEBV function

**FIG. 4.**
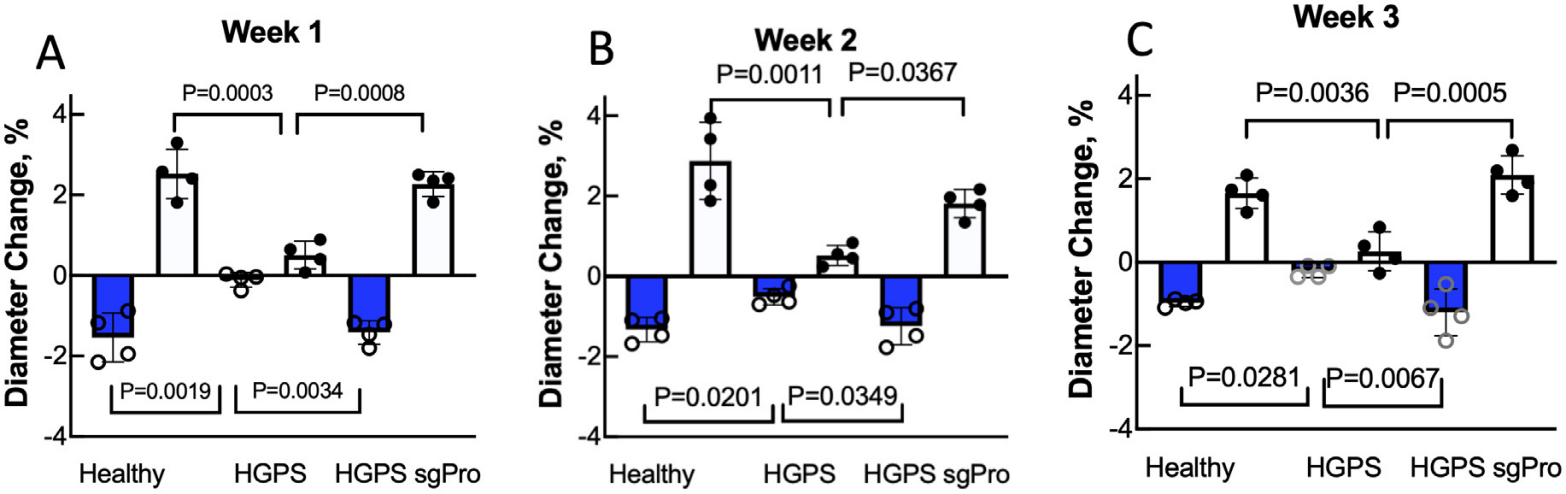
ABE7.10max treatment restores HGPS TEBV vasoactivity to healthy levels. Diameter change of healthy and HGPS TEBVs fabricated with viSMCs and viECs that were either untreated or treated with ABE7.10max + *LMNA* mutation-targeting single guide RNA (sgPro), perfused for 1 (a), 2 (b), or 3 weeks (c) and then diameters measured before and after exposure to 1 *μ*M phenylephrine (blue bars) or 1 *μ*M acetylcholine (white bars) for 5 min. Data presented as mean ± SD. N = 4 TEBVs per group. Results compared using one-way ANOVA followed by a post hoc Tukey's test. Time did not have a significant effect on measurements within each group.

Next, to determine whether the edited HGPS viSMCs and viECs would result in TEBV function and histology similar to healthy TEBVs, TEBVs were fabricated with healthy viSMCs and viECs, HGPS viSMCs and viECs, or sgPro viSMCs and viECs and then matured for 1–3 weeks. When tested after 1, 2, or 3 weeks of perfusion, HGPS TEBVs exhibited significantly reduced vasoactivity relative to TEBVs made with healthy viSMCs and viECs (Fig. 4). sgPro TEBVs exhibited significantly improved vasoconstriction and vasodilation that did not differ from healthy levels at all three timepoints (Fig. 4). Over the 3 weeks of culture, duration of perfusion did not have a significant effect on vasoconstriction or vasodilation within each group.

### Base editing restores HGPS TEBV protein expression and cell numbers while reducing extracellular matrix proteins

**FIG. 5.**
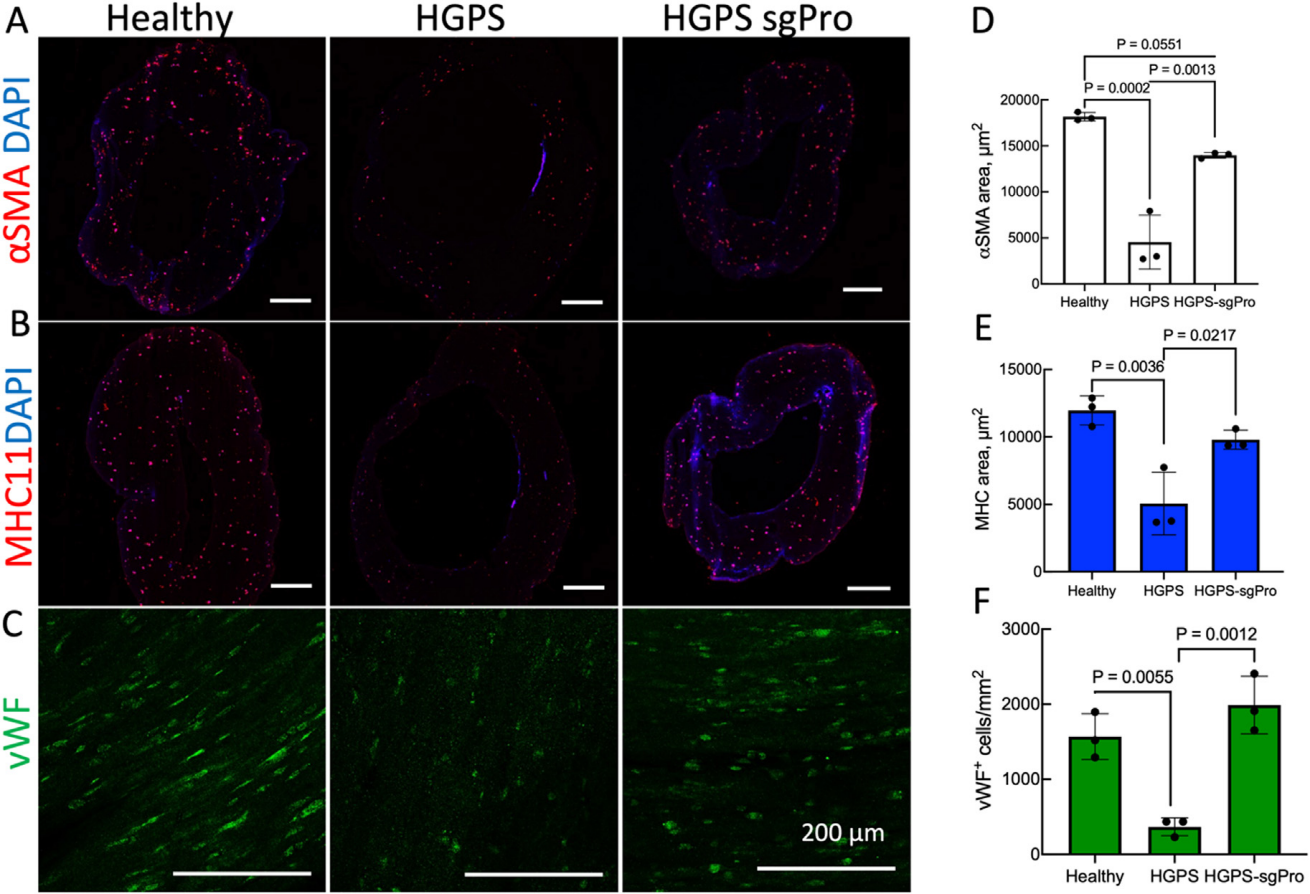
ABE7.10max treatment prevents SMC loss in HGPS TEBVs. Representative images of (a) α-smooth muscle actin (αSMA) and (b) myosin heavy chain 11 (MHC11) from cross sections of TEBVs fabricated with healthy cells, HGPS cells, or HGPS cells treated with ABE7.10max + *LMNA* mutation-targeting guide RNA (sgPro). Images in (a) and (b) are taken from 10 *μ*m cryosections of fixed TEBVs. (c) Representative images of *en face* sections of the endothelial surface of TEBVs stained with an antibody to vWF. Conditions are the same as in panels (a) and (b). Scale bar equals 200 *μ*m in all panels. (d) Quantification of αSMA-positive area from images in (a). (e) Quantification of MHC11+ area from images in (b). (f) Quantification of vWF-positive cells from images in (c). Data presented as mean ± SD. N = 3 TEBVs per group. Results compared using one-way ANOVA followed by post hoc Tukey's test.

HGPS TEBVs express lower levels of α-smooth muscle actin (αSMA) and myosin heavy chain 11 (MHC11), which are expressed in contractile smooth muscle cells, compared with healthy TEBVs [Figs. 5(a) and 5(b)], as we observed previously.^25^ HGPS TEBVs also expressed lower levels of endothelial protein von Willebrand Factor (vWF) [Fig. 5(c)]. Edited sgPro TEBVs displayed increased levels of αSMA, and MHC11 compared with untreated HGPS TEBVs, similar to healthy levels [Figs. 5(a), 5(b), 5(d), and 5(e)]. Likewise, edited sgPro viECs in TEBVs expressed vWF similar to levels in healthy TEBVs and greater than in HGPS TEBVs [Figs. 5(c) and 5(f)].

**FIG. 6.**
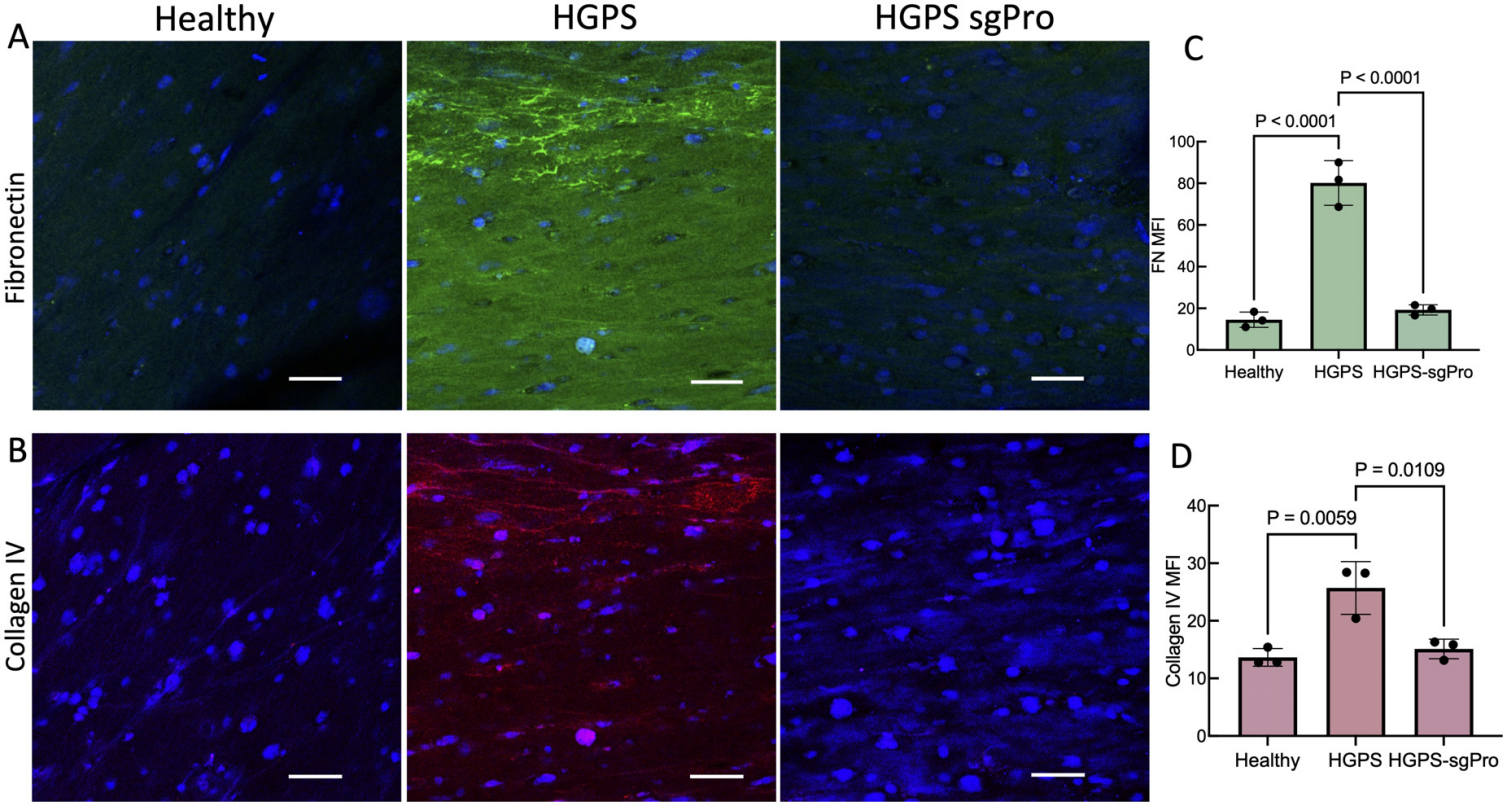
ABE7.10max treatment reduces ECM deposition in HGPS TEBVs. Representative images of (a) fibronectin (FN) and (b) collagen IV expression in TEBVs fabricated with healthy cells, HGPS cells, or HGPS cells treated with ABE7.10max + LMNA mutation-targeting guide RNA (sgPro). Images in (a) and (b) are longitudinal confocal images of stained whole TEBV samples. Scale bar 50 *μ*m. (c) Quantification of fibronectin mean fluorescence intensity (MFI) from images in (a). (d) Quantification of collagen IV MFI from images in (b). Data presented as mean ± SD. N = 3 TEBVs per group. Results compared using one-way ANOVA followed by a post hoc Tukey's test.

Consistent with two-dimensional (2D) results in viECs and viSMCs, HGPS TEBVs exhibited lower levels of viSMC DAPI-stained nuclei and Ki67 expression compared with healthy TEBV [Figs. S5(a) and S5(b)]. sgPro TEBVs restore higher levels of viSMC cell nuclei and Ki67 expression compared with HGPS TEBVs, similar to healthy levels as shown by the images and quantification of cell numbers (supplementary material, Figs. S5(a)–S5(d)]. HGPS TEBVs exhibited significantly higher expression levels of extracellular matrix proteins fibronectin and collagen IV, two proteins found in the fibrotic plaques of HGPS patients, compared with healthy TEBVs [Figs. 6(a)–6(d)]. Edited sgPro TEBVs exhibited significantly lower fibronectin and collagen IV expression that is not statistically significant from the corresponding levels in healthy TEBVs.

### Fraction of edited viSMCs increases over time in co-cultures of edited and unedited cells

**FIG. 7.**
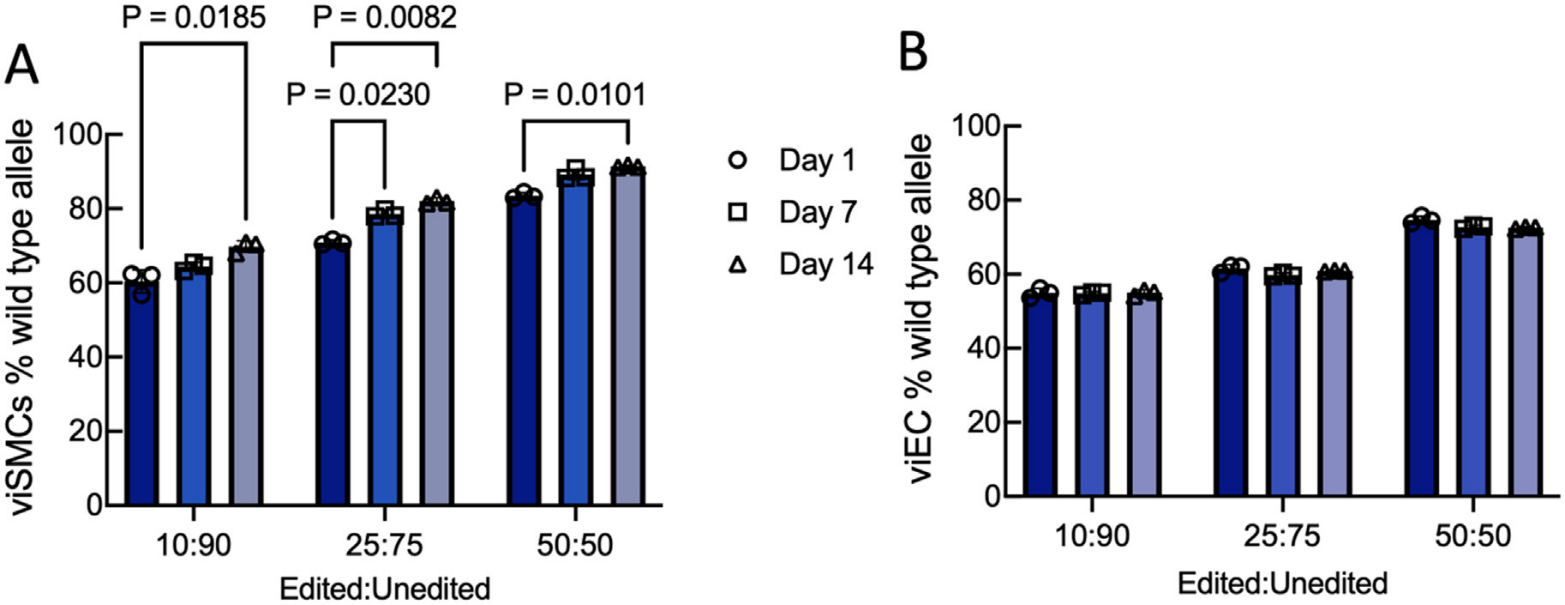
Fraction of edited viSMCs but not viECs increases over time in co-cultures of sgPro-edited and unedited HGPS cells. Percent wild-type allele at the *LMNA* 1824 C > T mutation site on days 1, 7, and 14 in co-cultures of sgPro-edited and unedited HGPS viSMCs (a) and viECs (b) at ratios of 10:90, 25:75, and 50:50 edited:unedited cells. Data presented as mean ± SD. N = 3 experiments per group. Results were analyzed using a two factor ANOVA (time and ratio of edited:unedited cells) and a Tukey's post hoc test.

*In vivo*, only a fraction of the cells is edited. Since HGPS cells are less proliferative than healthy cells [Figs. 2(c) and 2(g)],^29^ it is possible that edited cells may outcompete unedited cells, resulting in increased editing efficiency over time. To evaluate the potential for this, we co-cultured mixtures of edited sgPro and unedited HGPS viSMCs or viECs at three different ratios: 10% edited:90% unedited, 25% edited:75% unedited, or 50% edited:50% unedited. High throughput sequencing on days 1, 7, and 14 was used to evaluate the frequency of wild-type allele in each culture. Percent wild-type allele did not increase over time in mixed cultures of edited and unedited viECs at any of the ratios we tested [Fig. 7(a)], which was confirmed by a two factor ANOVA (time and ratio of edited:unedited cells). In contrast, percent wild-type allele increased significantly over 2 weeks in all three viSMC cultures. For the two factor ANOVA, both factors were significant, each with p < 0001. A post hoc Tukey's test showed that even when the edited cells only made up 10% of the population on day 1, significant increases in cell number were observed [Fig. 7(b)]. For the most part, bystander editing varied in proportion to the fraction of edited cells, and the percent of indels did not change with time (Fig. S6).

### Mixing edited and unedited HGPS cells in TEBVs identifies fraction of edited human cells needed to restore function

Since the cell culture experiments suggested improvement in edited viSMC cell number in mixed cultures over time, we sought to identify a minimum edited fraction of cells that resulted in improvement of the TEBV phenotype. We prepared TEBVS with the following ratios of edited to unedited viECs and viSMCs and perfused the TEBVs for three or 5 weeks: 25:75, 50:50, and 75:25. We chose higher ratios of edited cells than those tested in 2D culture to ensure that we could differentiate the responses from HGPS TEBVs. TEBVs prepared with healthy or HGPS cells served as positive and negative controls, respectively, although the healthy control TEBVs were not included in the 5-week experiment.

**FIG. 8.**
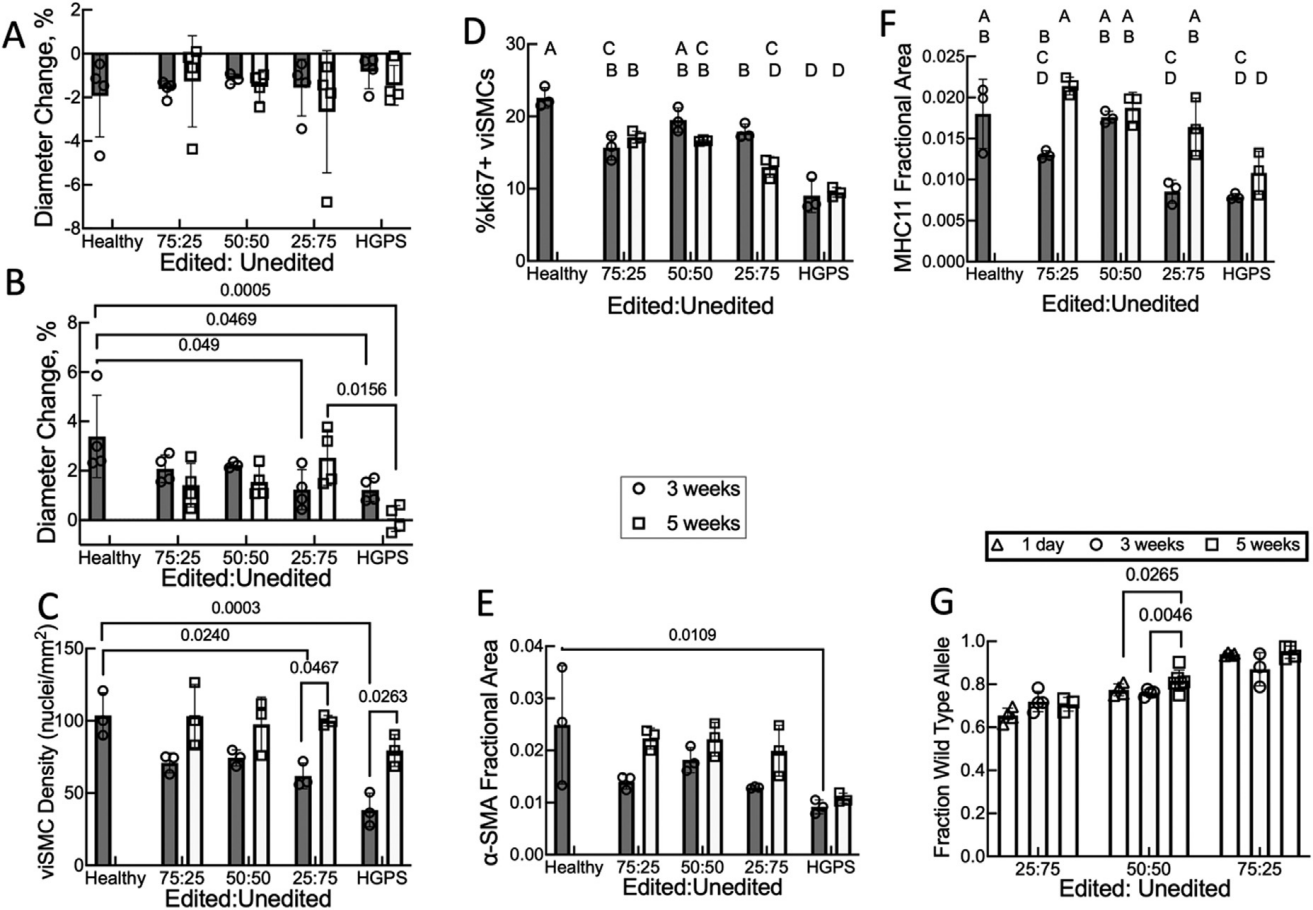
A ratio of edited:unedited HGPS cells in TEBVs above 50:50 improves viSMC proliferation and MHC11 levels after 3 or 5 weeks of perfusion. TEBVs were prepared with healthy or HGPS viSMCs and viECs as well as the following ratios of edited to unedited cells 75:25, 50:50, and 25:75. TEBVs were perfused for three or five weeks. To evaluate vascular function, diameters were measured before and after exposure to 1 *μ*M phenylephrine (a) for 5 min, followed by 1 *μ*M acetylcholine (b) for 5 min. TEBVs were flash frozen, and frozen sections stained for α-smooth muscle actin (α-SMA), myosin heavy chain 11 (MHC11), and DAPI-stained nuclei are shown in Fig. S7 for 3-week perfusion and Fig. S8 for 5-week perfusion. Quantification of viSMC nuclei density (c), percent Ki67-positive viSMC nuclei (d), α-SMA (e), and MHC11 (f). (g) Fraction of wild-type allele in TEBVs determined by high throughput sequencing. Data presented as mean ± SD, N = 4 TEBVs per group for vasoactivity and N = 3 TEBVs for immunostaining. Results compared by two-way ANOVA followed by post hoc Tukey's test. For panels (d) and (f), conditions not connected by same letter are significantly different. Exact p values are listed in supplementary material, Tables S1 and S2. For panel G, a one-way ANOVA was performed with a Tukey post hoc test.

Vasoconstriction was not significantly affected by the editing ratio [Fig. 8(a)], but vasodilation was reduced below healthy TEBV levels at a ratio of 25:75 at 3 weeks, and the healthy TEBV vasodilation at 3 weeks exceeded the HGPS vasodilation at 3 and 5 weeks [Fig. 8(b)]. Vasodilation in TEBVs with 50% and 75% edited cells was at an intermediate level that did not significantly differ from healthy or HGPS TEBVs. At 5 weeks, vasodilation in TEBVs with 25% edited cells increased and was significantly higher than TEBVs made with HGPS cells at the same timepoint [Fig. 8(b)]. This was matched by an increase in viSMC density in 25:75 edited TEBVs over 3–5 weeks, although the increase in αSMA and MHC11 area in these TEBVs did not reach statistical significance [Figs. 8(c), 8(e), and 8(f), supplementary material, Fig. S7(a) and S8(a)]. At 3 weeks, the viSMC density in TEBVs with 25% edited cells was lower than healthy TEBVs, while the viSMC density in TEBVs with 50% or 75% edited cells was intermediate between healthy TEBVs and HGPS TEBVs [Fig. 8(c)]. viSMC density did not significantly increase in 50% and 75% edited TEBVs from 3 to 5 weeks [Fig. 8(c)]. Surprisingly, viSMC density in HGPS TEBVs increased between 3 and 5 weeks of perfusion, although this did not result in increased vasoactivity or α-SMA and MHC11 staining [Figs. 8(a)–8(c), 8(e), and 8(f)].

The percentage of viSMCs positive for Ki67 were elevated above levels in HGPS but were below levels in healthy TEBVs [Fig. 8(d)]. Immunofluorescence images suggest differences in α-SMA and MHC11 staining (supplementary material, Figs. S7 and S8). Although αSMA immunostaining quantification was different between healthy and HGPS TEBVs, TEBVs made with mixing edited and unedited cells were intermediate and did not significantly differ from either group [Fig. 8(e)]. In contrast, at 5 weeks, immunostaining for MHC11 was significantly higher in TEBVs made with with 0% and 75% edited cells than in HGPS TEBVs and did not significantly differ from healthy TEBVs at 3 weeks [Fig. 8(f)]. These results suggest that at 3 weeks, a minimum level of 50% edited cells is needed for improvement in viSMC density and MHC11 expression. Over the next 2 weeks of perfusion, the viSMC density, vasoconstriction, and MHC11 expression in 25% edited TEBVs increased to levels that are similar to 50% and 75% edited TEBVs, for all three editing ratios, although not all of the phenotypic markers quite reached levels in healthy TEBVs.

To assess whether changes in the wild-type allele during the perfusion might explain some of the improvements in TEBV function and viSMC contractile protein and density, Sanger sequencing was performed on TEBVs after 1 day, 3 weeks, and 5 weeks of perfusion. TEBVs prepared with 25% and 75% edited cells did not exhibit any significant change in the wild-type allele over 5 weeks [Fig. 8(g)]. In contrast, for TEBVs prepared with 50% edited cells, the wild-type allele at 5 weeks was greater than at 1 day or 3 weeks. While the wild-type allele frequency with 25% or 50% edited cells at 1 day corresponded to the expected ratio (0.654 ± 0.034 (mean ± SD) vs an expected ratio of 0.625 for 25% editing and 0.774 ± 0.028 vs an expected ratio of 0.75 for 50% editing), the wild-type allele frequency with 75% edited cells was higher than expected (0.939 ± 0.007 vs an expected ratio of 0.875).

## DISCUSSION

Adenine base editing to correct the most common point mutation for HGPS reduces progerin levels and nuclear blebbing in HGPS fibroblasts and improves vascular SMC density, reduces adventitial fibrosis, and improves lifespan in progeroid mice.^21^ To evaluate further effects of ABE on human vascular cells, endothelial dysfunction, and other cardiovascular HGPS pathologies, we tested the maximum possible benefit of base editing on HGPS TEBVs by transducing HGPS iPSCs with ABE7.10max sgPro, an ABE designed to target the HGPS mutation in *LMNA* locus and revert it to the wild-type allele. To inform the level of editing that would be necessary to improve vascular function, we assessed TEBVs with mixtures of edited and unedited viECs and viSMCs. Editing HGPS iPSCs with ABE7.10max sgPro restored the wild-type allele at the HGPS mutation site while only causing limited bystander editing and indel frequency. These responses were maintained in the edited viECs and viSMCs, which displayed 99% wild-type allele with minimal bystander editing within the range reported in HGPS fibroblasts.^21^ ABE sgPro treatment also decreased the levels of nuclear blebbing typical of HGPS cells to healthy levels in both edited viECs and viSMCs. These results are consistent with published results in HGPS fibroblasts from different donors, which displayed 91%–97% mutation correction, <2.2% bystander editing, and decreased abnormal nuclear morphology in edited HGPS cells^21^ and results of direct ABE7.10max transduction in HGPS iPSC-derived ECs, which resulted in 96% mutation correction and decreased nuclear blebbing.^24^

Several studies have implicated oxidative stress, DNA damage, and senescence in HGPS fibroblasts,^30–32^ but few have studied these typical characteristics of endothelial dysfunction in HGPS endothelial cells. With a single treatment, base editing restored ROS, proliferation, and DNA damage to healthy levels in both viECs and viSMCs. These results indicate that at its maximum efficiency, ABE treatment is a promising method to alleviate negative characteristics of HGPS viECs and viSMCs that may contribute to vascular dysfunction.

Endothelial NO synthesis is critical for normal vascular function.^33^ Reduced NO synthesis is a key feature of endothelial dysfunction that is one of the earliest indicators of atherosclerosis.^34^ In HGPS, NO production is reduced by decreased *NOS3* gene expression [Fig. 3(b)] as well as *NOS3* threonine 495 phosphorylation.^24^ The ABE7.10max improves nitric oxide production in static HGPS viECs.^24^ In this study, base editing restored HGPS viEC flow-mediated NO production to healthy levels and improved the expression of key shear sensitive genes to healthy levels.

Features of atherosclerosis in HGPS involve SMC depletion, endothelial dysfunction, extracellular matrix production, vessel stiffening, calcification and inflammation, and fibrotic plaque formation (*25*, *46*, *54*). To test the maximum benefit of base editing on HGPS vessels, we fabricated and characterized TEBVs with edited viECs and viSMCs. Unedited HGPS TEBVs displayed significantly reduced vasoactivity compared with healthy TEBVs. Edited HGPS sgPro TEBVs restored vasoactivity to levels not significantly different from levels exhibited by healthy TEBVs, which was maintained for 3 weeks of perfusion. After 3 weeks of perfusion, unedited HGPS TEBVs expressed lower levels of smooth muscle cell proteins αSMA and MHC11 and much lower cell density than healthy TEBVs, consistent with the histology of arteries from HGPS patients.^35^ Base editing of HGPS cells led to normal levels of αSMA and MHC11 protein as well as improved SMC cell density and proliferation in TEBVs. Base editing also increased vWF expression in the endothelium of TEBVs. HGPS TEBVs expressed fibronectin and collagen IV, two proteins typically found in HGPS patients' fibrotic plaques,^5^ which were reduced to healthy levels in edited HGPS sgPro TEBVs. These results are consistent with the reported improved SMC retention in base edited HGPS mice.^21^

Thus, TEBVs made only with edited viECs and viSMCs indicate that if transduction led to all cells being edited, then function would be completely restored. To mimic *in vivo* conditions in which a fraction of the cells are edited,^21^ we made TEBVs with various ratios of edited to unedited cells. TEBVs made with 50% or more edited cells restored a number of critical vascular functions and increased smooth muscle cell number, and this improvement could be observed in the TEBVs after 3 and 5 weeks of perfusion. Interestingly, while TEBVs made with 25% edited cells had significantly lower vasodilation, viSMC density, and MHC11 expression than healthy TEBVs at 3 weeks, these levels increased at 5 weeks of perfusion, indicating an improvement in phenotype over time.

These results suggest that partial editing of vascular cells can improve blood vessel structure and function. While we did observe partial improvement of TEBV function with mixtures of edited and unedited cells after 3 and 5 weeks of perfusion, longer perfusion times may lead to further functional improvements and approach the complete vascular recovery observed after 6 months in HGPS mice treated with ABE.^21^ In addition, autocrine or paracrine effects may improve vascular function beyond what might be expected from editing alone. For example, edited ECs may secrete Ang2, which decreases HGPS SMC senescence and improves their contractile phenotype.^36^ Progerin levels and lifetimes vary among tissues.^37^ Increased production of ghrelin by the stomach or endothelium^38^ could increase removal of progerin by autophagy^39^ in those tissues with limited editing and long progerin lifetimes, such as smooth muscle.

This study also has some limitations. In general, iPSC-derived cells represent an immature phenotype, so their response may not fully replicate the response to editing primary cells. Characterization of the viECs and viSMCs in this study and our prior work^25,26^ showed that they can reproduce key vascular functions, although the level of vasoactivity is reduced relative to levels with primary cells.^25^ While TEBVs made with HGPS viECs replicate key changes to HGPS arteries including SMC loss, vessel thickening, vessel calcification, and increased extracellular matrix,^5^ the response with mixtures of edited and unedited HGPS ECs and SMCs derived from edited iPSCs may under-represent the changes to vessel wall structure and function. Another limitation of the model is the absence of an adventitia. Given that adventitial thickening is a feature of HGPS, future models should incorporate an adventitial layer to assess its response.

The third limitation is that we only studied one HGPS donor, a 2-year-old (HGPS003). We previously compared iPSC-derived vascular cells and the resulting TEBVs from the 2-year-old (HGPS003) and 8-year-old (HGADFN167) HGPS subjects in terms of function and phenotype^25^ and with and without treatment with Lonafarnib and Everolimus.^26^ The TEBV vasoactivity was similar for these two donors, although HGPS 003 had slightly more vasoconstriction and expression of myosin heavy chain and α-smooth muscle actin than HGADFN167.^25^ The response of viECs and viSMCs from cell lines from the two subjects was very similar in all the experiments we tested (ROS, proliferation, DNA damage, and shear stress response).^26^ Furthermore, despite the age difference between the subjects, we did not see an exacerbation of HGPS phenotype in the cells from the older subject. While the disease phenotype is similar between cell lines from the two subjects, it would be valuable to include evaluation of ABE correction in cells from the older HGPS subject in a future study, particularly for the mixing experiments that could better inform differences in responsiveness to the ABE treatment between cell lines from the two subjects.

Overall, the results show that a vascular microphysiological system can test the effectiveness of base editing to improve vascular function in HGPS. Further modification by increasing the SMC cell number and adding an adventitial layer can lead to closer fidelity to *in vivo* conditions. Subsequent studies can examine whether the TEBV system can replicate a more physiological treatment of the vessel wall cells by introduction of the ABE and guide RNA in AAV in the perfusion media after TEBV fabrication with HGPS cells. However, while AAV9 has a high *in vivo* transduction efficiency, the transduction efficiency in cultured cells is low, necessitating the use of other AAVs *in vitro*.^40^ With optimization of the AAV for *in vitro* studies, it is possible to assess whether editing can reverse the vascular features of the disease once the HGPS phenotype is established in this *in vitro* vascular microphysiological system. The model could then be used to test whether the improved vascular function reported in mice is due local changes within the vessel wall or whether paracrine factors act in concert with ABE to improve vascular function after editing.

## METHODS

### iPSC Cell Culture

iPSCs (Progeria Research Foundation, PRF) used in this study were healthy donor 168 clone CL2 and the HGPS lines 003, clones CL1C and CL1D, which contain the progerin mutation (c.1824 C > T) in Exon 11. The Duke University Health System Institutional Review Board determined that the use of human cells provided by PRF does not meet the definition of research involving human subjects. iPSCs were cultured with mTeSR Plus media (StemCell Technologies) on dishes coated with hESC-qualified Matrigel (BD Biosciences). For maintenance culture, iPSCs were passaged at with 0.5 mM EDTA (Invitrogen) when they reached 80%–90% confluency.

### Lentiviral vector cloning and production

The ABE7.10max-VRQR lentiviral vector, which contains the targeting guide RNA (sgPro) for the *LMNA* mutation, was prepared as previously reported.^21^ To clone the control ABE7.10max lentiviral vector with non-targeting guide RNA (sgNT),^27^ the sequence of sgPro and its U6 promoter was excised with restriction enzymes KpnI and NheI. The digested, linearized plasmid was then gel purified and ligated with sgNT-containing gene block (IDT) using T4 DNA ligase (NEB).

HEK239T/17 (ATCC CRL-11268) cells were maintained at 37 °C with 5% CO_2_ in antibiotic-free DMEM (Thermo Fisher) containing 10% (v/v) fetal bovine serum (Thermo Fisher). For lentivirus production, rapidly dividing HEK293T/17 cells were split 1:3. After changing the medium the next day, cells were transfected with FuGENE HD (Promega) according to the manufacturer's protocols. The transfection mix included 9 μg of the packaging genome of interest (transfer vector), 9 *μ*g of psPAX2, which encodes the viral packaging proteins, and 6 *μ*g of pVSV-G, which encodes the VSV-G envelope protein. FuGENE (70 μl) at room temperature was added to the transfection mix, and Opti-MEM was added to achieve a final volume of 1.5 ml per flask. The medium was collected after 2 days and spun at 3000 g for 15 min to remove any suspended cells. The supernatant was filtered (0.45 *μ*m PVDF) to eliminate all non-viral debris and then concentrated using Lenti-X Concentrator (TAKARA) according to the manufacture's protocol. The virus pellet was resuspended with Opti-MEM, and small aliquots in the PCR tubes were then flash frozen and stored in the −80 °C freezer.

### Lentiviral transduction of HGPS iPSCs

100 000 iPSCs were seeded into one well of a 6-well plate with 0.002 (v/v) lentivirus delivering ABE7.10max-VRQR.^21^ The lentivirus was removed after 24 h, and the media was refreshed. Two days later, 1 *μ*g/mL puromycin was added and replenished daily for 4 days. Selected iPSCs were then expanded and differentiated normally.

### Sanger & Next Generation Sequencing

Genomic DNA was collected from iPSCs, viSMCs, or viECs using the DNeasy Blood and Tissue Kit (Qiagen 69504). Genomic DNA was then sent to Genewiz for PCR amplification and Sanger Sequencing of a 501 bp region around *LMNA* c.1824.

For next generation sequencing (NGS), genomic DNA was amplified by qPCR using Phusion U Green Multiplex PCR Master Mix. To amplify the specific genomic region of interest, 25 *μ*l samples consisted of 0.5 *μ*M of forward and reverse primer, 1 *μ*l of genomic DNA extract, and 12.5 *μ*l of Phusion U Green Multiplex PCR Master Mix. The following PCR reactions were performed: 98 °C for 2 min followed by 28 cycles at 98 °C for 10 s, 61 °C for 20 s, and 72 °C for 30 s. A final 2 min extension at 72 °C was then performed. A secondary PCR reaction (PCR 2) of volume 25 *μ*l consisted of 0.5 *μ*M of each unique forward and reverse barcoding primer pair (Illumina), 1 *μ*l of unpurified PCR 1 reaction mixture, and 12.5 *μ*l of Phusion U Green Multiplex PCR 2× Master Mix. Barcoding PCR 2 reactions were performed as follows: 2 min at 98 °C followed by 12 cycles for 10 s at 98 °C, 20 s at 61 °C, and 30 s at 72 °C. A final 2 min at 72 °C extension was then performed.

PCR 2 products were pooled by common amplicons and purified by electrophoresis with a 1.5% agarose gel using a QIAquick Gel Extraction Kit (Qiagen) and eluted with 35 *μ*l of water. Gel-extracted, pooled PCR products are measured by fluorometric quantification (Qubit, Thermo Fisher Scientific) or qPCR (KAPA KK4824). Human *LMNA*-specific primers are as follows: Fwd 5′- **ACACTCTTTCCCTACACGACGCTCTTCCGATCTNNNN**ACCCCGCTGAGTACAACC-3′ and Rev 5′-**TGGAGTTCAGACGTGTGCTCTTCCGATCTNNNN**TCCTACCCCTCGATGACCAG-3′. High throughput sequencing was performed as described in Ref. 21.

MiSeq Reporter (Illumina) was used to demultiplex sequencing reads. Amplicon sequences were aligned to a reference sequence using CRISPResso2 (Ref. 41) in the batch mode with a window width that covers the entire protospacer sequence (20 nts).

### viSMC and viEC Differentiation^25,42^

iPSCs were cultured with 10 *μ*M Y-27632 (Tocris Bioscience). iPSCs were dissociated with Accutase and replated onto plates coated with Matrigel at 37 000 cells/cm^2^ for viSMC differentiation or 47 000 cells/cm^2^ for viEC differentiation. On the next day, to induce mesoderm, the cells were incubated for 3 days with N2B27 medium (1:1 mix of Neurobasal medium and DMEM/F12 with HEPES supplemented plus N2 supplement and B27 minus vitamin A (Gibco), 25 ng/mL BMP4 (PeproTech), and 8 *μ*M CHIR99021 (Cayman Chemical)).

To differentiate iPSCs to viSMCs, mesoderm induction media was changed to viSMC induction medium (N2B27 medium with 10 ng/mL PDGF-BB (PeproTech) and 2 ng/mL Activin A (PeproTech)) on day 4. Media was changed 1 day later. To induce a contractile SMC phenotype, on day 6, after detachment with Accutase, the cells attached to collagen-coated plates in viSMC media (N2B27 media supplemented with 2 ng/mL Activin A and 2 *μ*g/mL heparin (Sigma-Aldrich)). Media was changed every other day. For viSMC expansion, cells were cultured on collagen-coated plates and passaged at 80%–90% confluency with Accutase. viSMCs were used between passages 4 and 6.

To differentiate iPSCs to viECs, the cells were cultured in viEC induction media (Stempro-34 SFM medium (Thermo Fisher) supplemented with 200 ng/mL VEGF165 (Genscript) and 2 *μ*M forskolin (Sigma-Aldrich)) starting on day 4. On days 5, 6, and 7, conditioned media was collected and replaced with fresh viEC induction media. On day 7, purified viECs were obtained by magnetic activated cell sorting (MACS). The process involved dissociating the cells with Accutase and neutralizing with cold Stempro-34 SFM. Cells were centrifuged at 1000 rpm for 5 min and then washed with PBS containing 0.5% BSA and 2 mM EDTA (MACS buffer). Cells were resuspended at 0.125 × 10^6^ cells/*μ*L in MACS buffer. For each × 10^6^ cells 0.2 *μ*L of each, the following was added to the cell suspension: FcR blocking reagent and microbeads with CD31 and CD144 antibodies (Miltenyi Biotec). The mixture was incubated for 15 min on ice. Cells were rinsed once with MACS buffer and then resuspended in 1 ml MACS buffer and run through an LS column attached to a MACS separator (Miltenyi Biotec). Unattached CD31-CD144-cells were discarded. viECs positive for CD31+ and CD144+ were recovered as described previously.^43^

viECs were cultured in a 1:1 mixture of fresh Stempro-34 SFM and conditioned medium supplemented with 2 *μ*g/mL heparin. After passaging, viECs were cultured in viEC media (Stempro34-SFM supplemented with 50 ng/mL VEGF and 2 *μ*g/mL heparin) and 10 *μ*M SB431542, a TGF-β inhibitor that promotes viEC proliferation.

### viEC Parallel Plate Flow Studies

To apply physiological shear stresses, 33 000 cells/cm^2^ viECs were plated on collagen-coated slide flasks (Nunc). Once a confluent layer was achieved, the slides were inserted into a custom-designed parallel plate flow chamber and connected to a closed flow loop as previously described.^43^ Steady laminar flow was gradually increased to 12 dynes/cm^2^ to maintain cell attachment as described previously.^43,44^ After reaching 12 dynes/cm^2^, cells were maintained under these conditions for 24 h. At the end, viECs were removed, and DAF-FM fluorescence (for NO production) or RT-PCR was performed.

### RT-PCR and Primers

The RNeasy Mini Kit (Qiagen) was used to extract total RNA from viECs. The iScript cDNA Synthesis Kit (Bio-Rad) was used to reverse transcribe RNA into cDNA. RT-PCR was performed in triplicate using the iQ SYBR Green Supermix (Bio-Rad) and the CFX Connect Real-Time PCR Detection System (Bio-Rad). Human *GAPDH* (VHPS-3541, Real-Time Primers) served as an internal control. Primer sequences are listed in supplementary material, Table S3. For viECs exposed to shear stress, gene expression was normalized to healthy viECs under static culture. To account for variability in baseline expression under static conditions across experiments, a normalization factor for each experiment was obtained as the ratio of the global average of all conditions within each group to the average of each experiment.^45^

### DAF-FM DA Nitric Oxide Assay

After exposure to flow or static treatment, viECs were incubated in viEC media containing 1 *μ*M DAF-FM diacetate (Thermo Fisher) for 30 min at 37 °C and rinsed with PBS. The viECs were incubated in fresh media for 15 min to enable de-esterification of intracellular diacetates. Images at 20× magnification were obtained with a Zeiss 880 Airyscan inverted confocal microscope and analyzed for mean fluorescence intensity using FIJI.

### TEBV Fabrication, Treatment, and Testing

To fabricate TEBVs,^25,26^ 1.5 × 10^6^ viSMCs in 90 *μ*L of viSMC media were added to 7 mg/mL type 1 rat tail collagen (Corning) in 0.02M acetic acid. 10× DMEM (Sigma-Aldrich) was added to the collagen solution to obtain a 1:10 (v/v) ratio. To enable gelation, 1 M NaOH was added to raise the pH to 8.5. The solution was immediately injected into a custom fabrication chamber containing four channels (180 *μ*L gel solution per channel) containing annular tubes serving as a mandrel in each channel. After gelling for 30 min at 37 °C, the water was removed by plastic compression. Removal of the mandrels produced a cylindrical lumen in each TEBV. To prevent leakage, the ends of the TEBV were secured using custom PDMS clamps.

Next, TEBVs were endothelialized by resuspending 2 × 10^6^ viECs in 0.45 ml viEC media. 50 *μ*L viEC suspension was injected through each TEBV lumen. The TEBV chamber was sealed and placed on a rotator at 10 revolutions per hour for 40 min at 37 °C to enable a uniform attachment of endothelial cells. The chamber was then connected to a closed flow loop with a media reservoir containing 15 ml viSMC media and a peristaltic pump (Masterflex). A physiological shear stress of 6.8 dynes/cm^2^ was obtained by continuous flow at 0.5 ml/min per TEBV. The TEBVs were perfused for as long as 5 weeks, and the media was changed every 2 days.

Vasoactivity was measured at room temperature in the TEBV perfusion circuit by recording images of TEBV diameter using a stereoscope (AmScope) and ISCapture software. After 30 s of normal perfusion, phenylephrine (Sigma-Aldrich) at a final concentration of 1 *μ*M was perfused through the system to induce vasoconstriction. After 5 min, 1 μM acetylcholine (Sigma-Aldrich) was perfused through the system to induce vasodilation. TEBV diameter was measured from images taken at baseline (prior to phenylephrine addition), at 5 min after phenylephrine addition, and at 5 min after acetylcholine exposure. The diameter at each time point was determined by averaging values at four equidistant widths along the TEBV length using FIJI. Vasoconstriction equals the percent change in diameter from the initial diameter before and after 5 min of phenylephrine treatment. Likewise, vasodilation equals the percent change in diameter after 5 min of exposure to phenylephrine to the diameter before and after 5 min of acetylcholine treatment.

### Western Blotting

HGPS SMCs and ECs were dissociated with Accutase, pelleted, and then lysed using RIPA lysis buffer (Thermo Scientific) with 1% protease inhibitor (Cytoskeleton) for 30 min. Lysates were centrifuged at 4 °C for 15 min (15 000 RPM), and supernatant was collected. Relative protein amounts were quantified using Precision Red assay. Lysates were diluted to equal concentrations using RIPA lysis buffer with 1% protease inhibitor and then mixed with Laemmli Sample Buffer containing 5% 2-mercaptoethanol (Bio-Rad) in a 3:1 ratio. Lysates were heated at 95 °C for 5 min and separated by SDS-PAGE on a 4%–20% polyacrylamide gel (Bio-Rad) under 125 V for 1 h. Protein was transferred to a polyvinylidene difluoride (PVDF) membrane using 350 mA for 1 h. Membranes were blocked for 1 h in Tris-buffered saline (Bio-Rad) with 0.1% Tween-20 (Bio-Rad) and 5% bovine serum albumin (Sigma-Aldrich). After blocking, primary antibodies were added to the membranes overnight at 4 °C, followed by a 1 h incubation with secondary antibodies at room temperature. The following antibodies were used: anti-lamin A/C (Abcam, 4C11 Clone or Mab 3211; Millipore, 1:1000), GAPDH (Santa Cruz Biotechnology, 0411 Clone, 1:500), goat anti-mouse secondary (Thermo Scientific, 1:5000), and goat anti-rabbit secondary (Vector Laboratories, 1:5000). Prior to imaging, membranes were incubated for 2–3 min in Clarity Western Peroxide Reagent (Bio-Rad). Images were taken using a ChemiDoc Imaging System (Bio-Rad).

### Immunofluorescence Staining

For 2D samples, cells were incubated with 4% paraformaldehyde for 15 min at room temperature, washed three times with PBS, permeabilized with 0.1% Triton-X for 5 min at room temperature, rinsed with PBS three times, incubated in 10% goat serum in PBS for 1 h at room temperature, and, finally, incubated with diluted primary antibody in 10% goat serum overnight at 4 °C. Cells were washed with PBS three times, incubated with an Alexa Fluor-labeled secondary antibody at 1:500 in 10% goat serum for 2 h at room temperature, and washed three times with DPBS. Nuclei were visualized by staining with Hoechst 33342 at 1:1000 in DPBS at room temperature for 5 min.

TEBVs attached to the perfusion chamber were fixed in 10% formalin for 10 min. After removal from the chamber, the TEBVs were placed in a 6 well-plate and further incubated in 10% formalin for an additional 50 min. TEBVs were washed with DPBS three times and cut into sections. One section of each TEBV was frozen in Optimum Cutting Temperature (OCT) compound (Tissue-Tek) in liquid nitrogen and stored at -80 °C until being sectioned into 10 *μ*m sections using a cryostat. The rest of each TEBV was cut *en face* to view the viECs. TEBV whole sections and cryosections were permeabilized by incubation with 0.1% Triton-X for 10 min. Samples were washed three times with DPBS and incubated in 10% goat serum in PBS for 30 min at room temperature (cryosections) or overnight at 4 °C (whole TEBV sections). To block nonspecific binding, cryosections and whole TEBV sections were stained with primary antibody in 10% goat serum overnight at 4 °C. Samples were washed three times with PBS and then incubated with secondary antibody and Hoechst 33342 in 10% goat serum for 2 h at room temperature. Samples were washed three times with PBS and mounted onto glass slides with FluorSave (Millipore). En face TEBV sections, TEBV sections, and 2D samples were imaged using a Zeiss 880 inverted confocal microscope at 20× magnification and analyzed using FIJI.

Primary antibodies used in this study were the following: rabbit anti-αSMA (Abcam, 1:200), rabbit anti-ki67 (Abcam, 1:200), rabbit anti-MHC11 (Abcam, 1:100), mouse anti-vWF (Abcam, 1:200), mouse anti-fibronectin (Abcam, 1:200), and rabbit anti-collagen IV (Abcam, 1:200). Secondary antibodies used were Alexa Fluor 594 goat anti-rabbit and Alexa Fluor 488 goat anti-mouse (Thermo Fisher, 1:500). Hoechst 33342 was used at 1:1000. Product numbers for all antibodies are provided in supplementary material, Table S4.

### Statistics

Statistical Analysis was performed using JMP Pro 14 (SAS). Data were analyzed using a one- or two-way ANOVA and post hoc Tukey tests for pairwise comparisons. For time-dependent assays on the same sample, repeated measures ANOVA was used. Results were plotted using GraphPad Prism. Data are represented as mean ± SD with N = number of TEBVs or number of independent experiments for 2D cell culture experiments; p ≤ 0.05 was considered significant.

## SUPPLEMENTARY MATERIAL

See the supplementary material for an outline of the experimental procedures, Western blot demonstrating elimination of progerin protein after editing, editing efficiency in viSMCs and viECs, DNA damage in HGPS viSMCs and viECs after editing, restoration of viSMCs in edited HGPS TEBVs, bystander editing in HGPS viECs and viECs, and the effect of the cell ratio of edited:unedited HGPS cells on SMC markers in TEBVs.

## Data Availability

High throughput DNA sequencing FASTQ files are available from the National Center of Biotechnology's Information Sequence Read Archive under BioProject (PRJNA1148490).Other data that supports this study are available at the Duke Digital Repository https://research.repository.duke.edu withthe following DOI: https://doi.org/10.7924/r4pg1xv1b.
